# Identification of Host Micro RNAs That Differentiate HIV-1 and HIV-2 Infection Using Genome Expression Profiling Techniques

**DOI:** 10.3390/v8050121

**Published:** 2016-05-02

**Authors:** Krishnakumar Devadas, Santanu Biswas, Mohan Haleyurgirisetty, Viswanath Ragupathy, Xue Wang, Sherwin Lee, Indira Hewlett

**Affiliations:** Laboratory of Molecular Virology, Division of Emerging and Transfusion Transmitted Diseases, Center for Biologics Evaluation and Research, Food and Drug Administration, 10903 New Hampshire Ave, Silver Spring, MD 20993, USA; Santanu.Biswas@fda.hhs.gov (S.B.); Mohan.Haleyurgirisetty@fda.hhs.gov (M.H.); Viswanath.Ragupathy@fda.hhs.gov (V.R.); Xue.Wang@fda.hhs.gov (X.W.); Sherwin.Lee@fda.hhs.gov (S.L.)

**Keywords:** host miRNAs, HIV-1 and HIV-2 infection, modulation of host factors

## Abstract

While human immunodeficiency virus type 1 and 2 (HIV-1 and HIV-2) share many similar traits, major differences in pathogenesis and clinical outcomes exist between the two viruses. The differential expression of host factors like microRNAs (miRNAs) in response to HIV-1 and HIV-2 infections are thought to influence the clinical outcomes presented by the two viruses. MicroRNAs are small non-coding RNA molecules which function in transcriptional and post-transcriptional regulation of gene expression. MiRNAs play a critical role in many key biological processes and could serve as putative biomarker(s) for infection. Identification of miRNAs that modulate viral life cycle, disease progression, and cellular responses to infection with HIV-1 and HIV-2 could reveal important insights into viral pathogenesis and provide new tools that could serve as prognostic markers and targets for therapeutic intervention. The aim of this study was to elucidate the differential expression profiles of host miRNAs in cells infected with HIV-1 and HIV-2 in order to identify potential differences in virus-host interactions between HIV-1 and HIV-2. Differential expression of host miRNA expression profiles was analyzed using the miRNA profiling polymerase chain reaction (PCR) arrays. Differentially expressed miRNAs were identified and their putative functional targets identified. The results indicate that *hsa-miR 541-3p*, *hsa-miR 518f-3p*, and *hsa-miR 195-3p* were consistently up-regulated only in HIV-1 infected cells. The expression of *hsa-miR 1225-5p*, hsa-miR 18a* and *hsa-miR 335* were down modulated in HIV-1 and HIV-2 infected cells. Putative functional targets of these miRNAs include genes involved in signal transduction, metabolism, development and cell death.

## 1. Introduction

Human immunodeficiency virus type 1 and 2 (HIV-1 and HIV-2) are closely related retroviruses that share 30%–60% overall genetic homology and similar structural and genomic organization [1]. In addition to structural similarities, these two related viruses exhibit functional similarities like cluster of differentiation 4 (CD4) binding, T-cell and mononuclear cell tropism, viral transmission, replication and pathogenesis [2,3,4]. However, in spite of the similarities between HIV-1 and HIV-2, several studies have shown that the disease outcomes presented by these two viruses are distinct [5]. HIV-2 is less transmissible than HIV-1 and the disease free survival time in HIV-2 infected patients is longer when compared to HIV-1 infected patients [5,6]. Furthermore, progression to immunodeficiency is less frequent with HIV-2 infection than with HIV-1 infection, even though the few who progress to immunodeficiency cannot be distinguished clinically from HIV-1-infected patients [7,8,9].

The exact mechanisms contributing to these distinct clinical outcomes in infections with the HIV-1 and HIV-2 viruses are not completely understood. One determinant of these differences may be a lower viral load observed in HIV-2 infected patients [10]. Studies have proposed that HIV-2 infection causes lower rates of T-cell activation and enhanced virus-specific immune responses leading to viral control in HIV-2 infections [11]. HIV-specific CD4+ and CD8+ T cell responses and high titers of broadly neutralizing antibodies found in HIV-2 infected individuals may lead to increased immunological control of HIV-2 replication [8,12,13]. In addition to the immune response induced by HIV-2 that might account for these differences in clinical outcomes, additional cellular factors may also be involved. Our published studies have shown significant variations in *in vitro* cytopathic effects following infection with HIV-1 and HIV-2. Preliminary findings using peripheral blood mononuclear cells (PBMC) or Jurkat cells infected with HIV-1 or HIV-2 indicated that HIV-1 infection generated more reactive oxygen species (ROS), increased the expression of a larger number of molecules involved in cell signaling such as p47, p38α, c-Jun N-terminal kinases (JNK), c-Yes, total protein kinase C (PKC), and decreased the expression of molecules such as p38β, extracellular receptor kinases (ERK)1/2, X-linked inhibitor of apoptosis protein (XIAP) leading to increased cell death by apoptosis relative to HIV-2 infection. We also found that HIV-1 induced more autophagic death in Jurkat cells relative to HIV-2 [14,15,16,17]. These results indicate that HIV-1 and HIV-2 may interact differently with host regulatory factors and modulate viral life cycle and disease progression.

The host cell upon infection by HIV undergoes many changes during active virus replication. These changes are manifested in multiple pathways like signal transduction, cell cycle progression and apoptosis and are mediated by proteins and mRNAs. MicroRNAs (miRNAs) constitute an additional class of molecules modulated by viral infection. MiRNAs are small non-coding RNA molecules which function in transcriptional and post-transcriptional regulation of gene expression. MiRNAs regulate gene expression through imperfect base pairing to complementary sites (miRNA binding sites) in corresponding target genes. The predominant mechanism of gene silencing involves translational repression and mRNA cleavage or alteration of mRNA stability. Since the silencing mechanism does not involve perfect complementary base-pairing, multiple genes can be targeted for repression by a single miRNA [18]. Recent studies suggest that HIV infection can alter host miRNA expression and control viral replication [19,20,21,22,23,24,25,26,27,28,29,30,31]. Furthermore, studies report that miRNAs encoded by HIV-1 and other viruses can act on cellular targets modulating viral replication and infectivity [32,33,34,35].

The aim of this study was to elucidate the differential expression profiles of host miRNAs in cells infected with HIV-1 and HIV-2 in order to identify potential differences in virus-host interactions between HIV-1 and HIV-2. Differential expression of host miRNA expression profiles was analyzed using the miRNA profiling polymerase chain reaction (PCR) arrays. Differentially expressed miRNAs were identified and their putative functional targets identified. The results indicate that *hsa-miR 541-3p*, *hsa-miR 518f-3p*, and *hsa-miR 195-3p* were up-regulated only in HIV-1 infected cells. While the expression of hsa-miR 18a*, *hsa-miR 499-3p* and *hsa-miR 335* were down modulated both in HIV-1 and HIV-2 infected cells, no miRNAs were found to be consistently modulated in HIV-2 infected cells. Putative functional targets of these miRNAs include genes involved in signal transduction, metabolism, development and cell death. These observations will further our understanding of the differences in the biological pathways involved in HIV-1 and HIV-2 infection and pathogenesis, leading to the identification of novel biomarkers that predict severity of disease progression.

## 2. Materials and Methods

### 2.1. Peripheral Blood Mononuclear Blood Cells Isolation and Cell Culture

Buffy coats from individual seronegative donors were provided by the National Institute of Health (NIH) Blood bank. Informed consent was obtained from all of the healthy, normal blood donors according to the ethical principles of international ethical guidelines for biomedical research involving human subjects. A categorical exemption is in place for Center for Biologics Evaluation and Research (CBER)/Food and Drug Administration (FDA) for experimental studies by CBER/ FDA researchers using existing, deidentified samples of blood and/or blood products originally obtained under the NIH institutional review board (IRB) approved protocol and consent form 99-CC-0168. This study was approved by the FDA IRB. Written informed consent to participate in a clinical research study involving the collection and distribution of blood components from healthy donors for *in vitro* research use and for publication of this Case Report and any accompanying images was obtained from healthy, normal blood donors according to the ethical principles of international ethical guidelines for biomedical research involving human subjects. This study was approved by the NIH ethics committee (study number: 99-CC-0168, principal investigator: Susan F. Leitman, MD). A single step Ficoll-Hypaque density gradient centrifugation method was used for isolating PBMC from buffy coats. The PBMC were stimulated with 10 μg/mL of -Phytohemagglutinin (PHA) for 72 h by culturing in complete Roswell Park Memorial Institute (RPMI)-1640 media (RPMI medium containing 10% heat inactivated fetal bovine serum (FBS), 1% penicillin-streptomycin, 10 U/mL interleukine-2 (IL-2)), and were cultured for an additional 24 h in complete RPMI media prior to infection. Jurkat cells were cultured in complete RPMI-1640 media.

### 2.2. Viruses and Infection

Jurkat cells were seeded at 1 × 10^6^ cells/mL for 24 h, and infected with known amounts (10 ng/mL p24/p27 units per 10^6^ cells) of HIV-1 (MN) [36,37] and HIV-2 (ROD) and cultured for different days as indicated. PBMC cells were infected with known amounts (5 ng/mL p24/p27 units per 10^6^ cells) of HIV-1 (MN) and HIV-2 (ROD). Two hours post-infection, tubes were washed with PBS to remove un-adsorbed virus and cells were fed with complete RPMI (RPMI 1640, 10% FBS, 1% penicillin–streptomycin for Jurkat cells and supplemented with IL-2 for PBMC) and cultured in T75 flask. The cells were harvested at different time points as indicated. HIV-1 and HIV-2 replication was quantitated by Alliance HIV-1 p24 Antigen ELISA Kit (Perkin Elmer, Waltham, MA, USA) and RETRO-TEK SIV p27 Antigen ELISA Kit (ZeptoMetrix, Buffalo, NY, USA).

### 2.3. RNA Extraction

Total RNA was extracted from infected and uninfected cells using miRNeasy total RNA isolation kit (QIAGEN, Valencia, CA, USA) according to the manufacturer’s protocol. Infected and uninfected cells were lysed with 700 μL of QIAzol lysis reagent. DNA was sheared using the QIAshredder columns (QIAGEN) and total RNA was extracted from this lysate using the RNeasy^®^ Mini column (QIAGEN). Total RNA was estimated by spectrophotometry using the Nanodrop 1000 (Thermo Fisher, Waltham, MA, USA).

### 2.4. miRNA Profiling Using RT^2^ MicroRNA PCR Array

Qiagen RT^2^ microRNA PCR Array system (Cat#MAH200, based on miRBase version 14), an optimized real-time PCR assay was used for miRNA profiling studies, which allows the simultaneous detection of 352 miRNAs, representing most functional miRNAs, as well as appropriate control miRNAs and RNA quality controls. Extracted total RNA including miRNAs (20 ng/μL concentration) was first reverse transcribed into cDNA using the miScript II RT kit following manufacturer’s recommendations (QIAGEN). One ng cDNA per well was then mixed with SYBR Green PCR Master Mix and placed into a 96-well PCR-array plate containing a panel of 88 mature miRNAs sequences for Real-time PCR analysis on an Applied Biosystems 7500 Real Time PCR system. Relative amounts were calculated by the ΔΔCT method.

### 2.5. PCR Array Data Analysis

The expression of individual miRNA was analyzed using Ct values obtained with a threshold of 0.2. All array plates were tested with endogenous controls, RT negative controls, and genomic DNA contamination controls. Ct values of 35 or greater for any particular miRNA in either the control or the experimental samples was excluded from the analysis and marked as undetectable or undetermined. The Ct values that passed through these stringent criteria were uploaded into the Qiagen/SABiosciences software (RT^2^ Profiler PCR Array Data Analysis) and the fold change calculated for each miRNA.

### 2.6. Validation of Differentially Expressed miRNA

Validation of miRNA expression was performed by real-time PCR (RT-PCR) experiments. Based on the data analyses, a subset of miRNAs was selected for validation by RT-PCR using specific primers and probes (Applied Biosystems, Waltham, MA, USA). We used total RNA samples from five independent donors to validate the high throughput PCR array results. Briefly, miRNA reverse transcription was done with the Taqman miRNA reverse transcription kit (Applied Biosystems); using 15 mM dNTPs, MultiScribe™ Reverse Transcriptase 50 U, 1.5 μL reverse transcription buffer, RNase inhibitor 3.8 U and 3 μL of 5 × RT primers for miRNA specific. Reverse transcription was carried out using the following conditions: 30 min at 16 °C, 42 °C for 30 min and 85 °C for 5 min. Real-time PCR was carried out using the following conditions: 10 min at 95 °C, followed by 40 cycles at 95 °C for 15 s and 60 °C for 1 min. The results were expressed as n-fold difference in expression of miRNA of interest relative to endogenous controls (RNU44) for infected and uninfected samples; n-fold=2^ ^− (ΔCt infected − ΔCt uninfected)^.

### 2.7. miRNA Targeted Gene Prediction

We utilized miRNA target gene prediction with the MirTarget2 prediction tool in miRDB database version 4.0. We selected top ten genes with a highest target score to validate the interaction between host genes and differentially expressed miRNA.

### 2.8. Quantification of Predicted Target Genes by RT-PCR

To validate the predicted miRNA target genes, we tested the expression of selected genes by RT-PCR using total RNA samples isolated from five independent donors. Quantitative RT-PCR was performed using the two-step RT-PCR with SYBR Green and ROX (Invitrogen, Waltham, MA, USA) according to manufacturer’s protocols. Total RNA was reverse transcribed with Superscript^®^ III First-Strand Synthesis SuperMix (Invitrogen). SYBR Green PCR mix (Invitrogen) and gene specific primers for real-time PCR were used for amplification. The program for thermal cycling was 20 s at 95 °C followed by 40 cycles of 3 s at 95 °C, 30 s at 57 °C. Glyceraldehyde-3-phosphate dehydrogenase (*GAPDH*) was used as an endogenous control for each donor. Each sample was run in triplicate to ensure accurate fold change estimation. Relative gene expression from each donor RNA was calculated from Ct values for the gene of interest and *GAPDH*. The Ct value of the gene-of interest was normalized to the Ct value of *GAPDH* and the final results expressed as n-fold difference in expression of gene-of-interest relative to *GAPDH* gene for infected and uninfected samples.

PBMCs were washed 3 times with ice cold PBS. The PBMCs were lysed with radioimmunoprecipitation assay (RIPA) buffer (Thermo Scientific cat # 89901, Waltham, MA, USA) and proteins were isolated by centrifugation. Total protein was quantitated using Pierce® BCA protein reducing agent compatible assay kit (Thermo Scientific). A total of 10 μg protein/lane was heated at 70 °C for 10 min in the NuPAGE^®^ LDS sample buffer and NuPAGE^®^ reducing agent and loaded on to a NuPAGE^®^ Bis-Tris Gel (4%–12%) (Life Technologies, Inc., Waltham, MA, USA). The separated proteins were transferred onto Amersham Hybond ECL Nitrocellulose membrane according to the manufacturer’s protocol. Proteins were detected using primary mouse monoclonal cullin 2 (CUL-2) antibody (Santa Cruz Biotechnology, Inc., Dallas, TX, USA), Cat # sc-166506), mouse monoclonal ubiquitin carrier protein 9/ubiquitin conjugating enzyme E2 I (UBC9/UBE2I) antibody (Santa Cruz Biotechnology, Inc., Cat # sc-271057), mouse monoclonal hook microtubule-tethering protein 3 (HOOK3) antibody (Santa Cruz Biotechnology, Inc., Cat # sc-398924), mouse monoclonal β-actin antibody (Santa Cruz Biotechnology, Inc., Cat # sc-47778) and rabbit anti organic anion transporter M1/solute carrier organic anion transporter family member 4C1 (OATP-H/SLCO4C1) polyclonal antibody (Santa Cruz Biotechnology, Inc., Cat # sc-368196). Proteins were visualized using an enhanced chemiluminescence (ECL) Western blotting analysis system (GE Healthcare, Pittsburgh, PA, USA).

## 3. Results

### 3.1. PCR-Array and Differential miRNA Expression Analysis

In our studies, the expression profile of 352 host cellular miRNAs was assessed in PBMC isolated from three independent donors productively infected with HIV-1 (MN) or HIV-2 (ROD) (Table 1) to identify putative biomarkers of infection that could differentiate between the two viruses.

**Figure 1:**
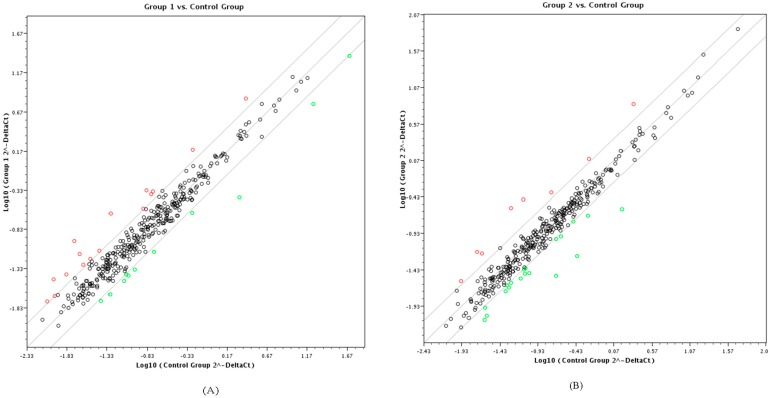
Scatter plot of differentially expressed miRNAs generated from QIAGEN web-based polymerase chain reaction (PCR)-array data analysis software. Group 1 represents human immunodeficiency virus (IV)-1 infected peripheral blood mononuclear cells (PBMC), Group 2 represents HIV-2 infected PBMC and control group represents mock infected (no virus) PBMC. Plot shows the microRNA (miRNA) expression in (**A**) HIV-1 infected PBMC *vs*. control PBMC and (**B**) HIV-2 infected PBMC *vs*. control PBMC. Red (**o**) circles represent upregulated and green (**o**) circles represent down regulated miRNAs with an expression level of greater than 2 fold upregultaion or downregulation respectivelly.

Differential expression of host miRNA expression profiles were analyzed using the miRNA profiling PCR arrays (QIAGEN/SA Biosciences). The miRNA PCR arrays represent a large panel of the most relevant miRNAs in the human genome (as annotated by the Sanger miRBase Release 14) and is designed to analyze miRNA expression using real-time PCR. The results of the expression profile of 352 host cellular miRNAs indicate that infection with HIV-1 and HIV-2 differentially regulated the expression of several miRNAs. The miRNA expression levels in HIV-1 and HIV-2 infected cells were compared with miRNA expression in uninfected PBMC cells, and internal controls were used for normalization and calculation of fold differences. The scatter plot of the differentially expressed miRNAs (Figure 1) indicates that the fold differences ranged between 2- and 7-fold for a number of miRNAs.

**Figure 2:**
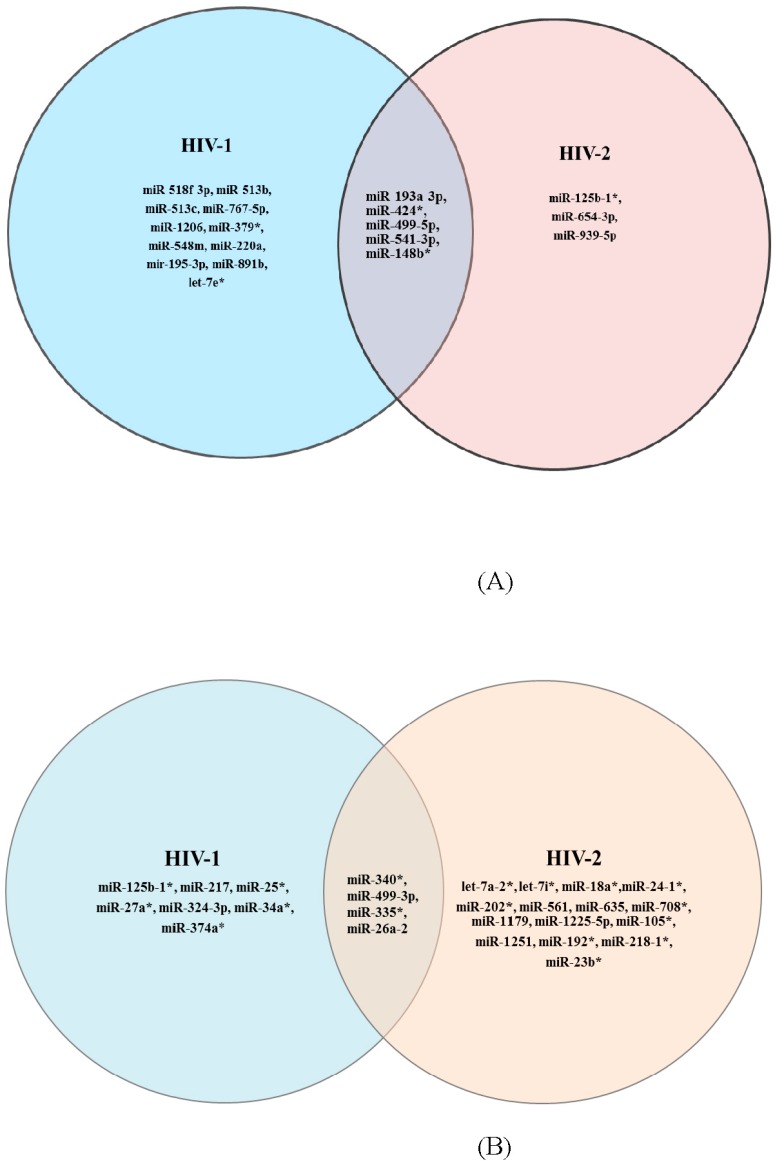
Venn diagram of differentially expressed miRNAs generated from QIAGEN web-based PCR-array data analysis software illustrating the overlap of upregulated or down regulated miRNAs between HIV-1 infected PBMC *vs.* control and HIV-2 infected PBMC *vs.* control. (**A**) Sixteen miRNAs showed greater than 2 fold upregulation in HIV-1 infected PBMC, whereas eight miRNAs showed upregulation in HIV-2 infected PBMC. Five miRNAs (miR-193a-3p, miR-424*, miR-499-5p, miR-541-3p and miR-148b*) were commonly upregulated in both HIV-1 and HIV-2 infected PBMC; (**B**) Eleven miRNAs demonstrated less than 2 fold downregulation in HIV-1 infected PBMC cells and nineteen miRNAs demonstrated less than 2 fold downregulation in HIV-2 infected PBMC cells respectively. Only four miRNAs (miR-340*, miR-499-3p, miR-335* and miR-26a-2) were commonly downregulated in both HIV-1 and HIV-2 infected PBMC cells.

Among the 352 miRNAs tested, 16 miRNAs were upregulated in HIV-1 infected PBMC and eight miRNAs were upregulated in HIV-2 infected PBMC (Figure 2) by more than 2-fold compared to uninfected control cells. Similarly, 11 miRNAs were downregulated in HIV-1 infected PBMC cells and 19 miRNAs were downregulated in HIV-2 infected PBMC cells (Figure 2) compared to uninfected controls. The Venn diagram (Figure 2) depicts miRNAs that are commonly upregulated or downregulated in both HIV-1 and HIV-2 infected cells.

**Figure 3:**
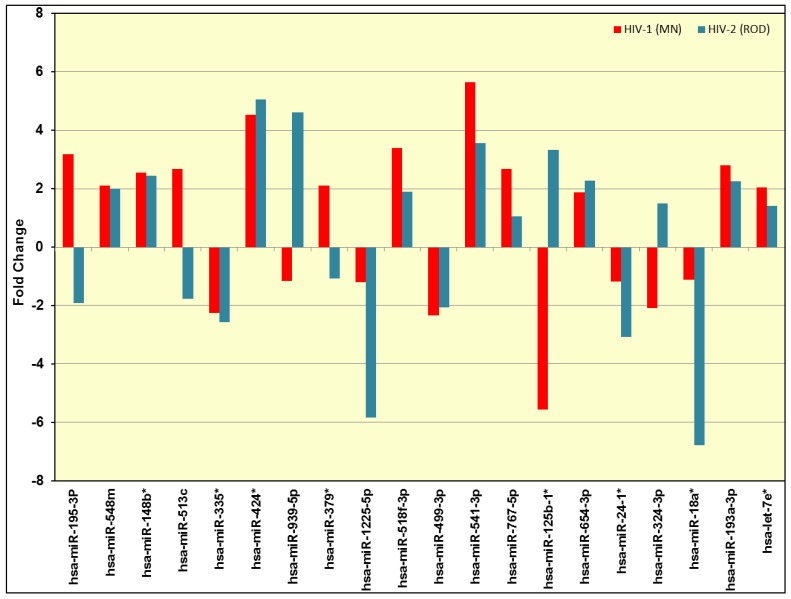
Identification of differentially expressed miRNAs in HIV-1 infected PBMC *vs.* control PBMC and HIV-2 infected PBMC *vs*. control PBMC using real-time PCR. We identified 20 differentially expressed miRNAs in both HIV-1 and HIV-2 infected PBMC. Red bars indicate the miRNA expression levels in HIV-1 infected PBMC and blue bars denote the miRNA expression levels in HIV-2 infected PBMC.

The results indicate that five miRNAs are commonly upregulated in both HIV-1 and HIV-2 infected cells and four miRNAs were commonly downregulated in both HIV-1 and HIV-2 infected cells. Out of the 45 differentially expressed miRNAs (Table 2), 20 differentially expressed miRNAs in HIV-1 and HIV-2 infected cells were selected for further analyses (Figure 3).

### 3.2. Validation of miRNA Expression by RT-PCR

**Figure 4:**
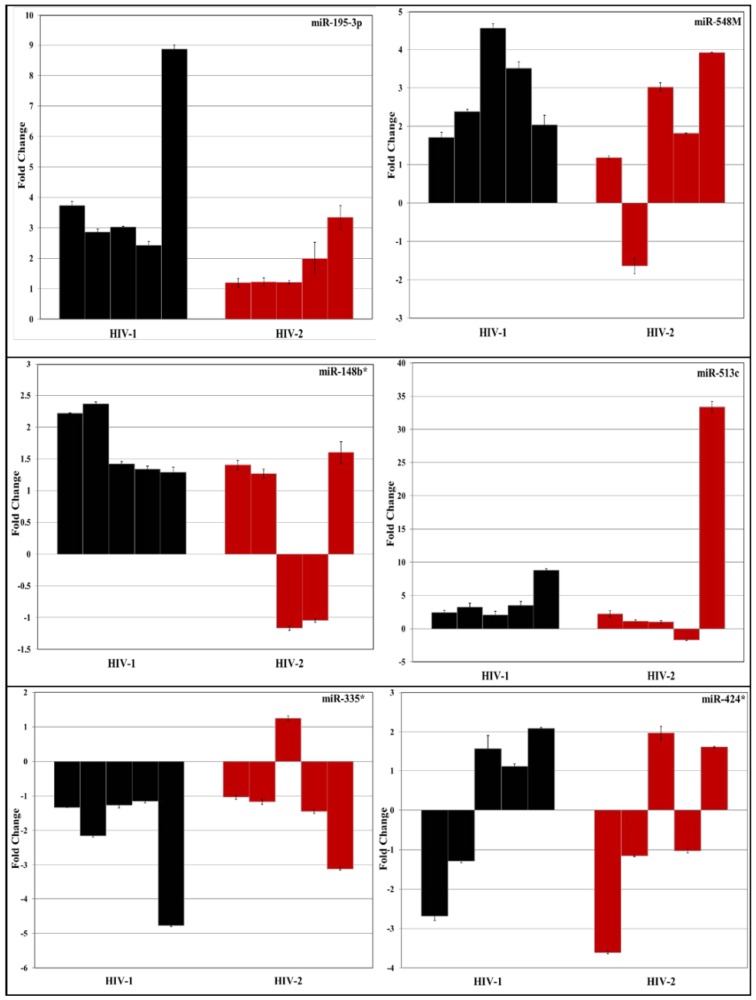

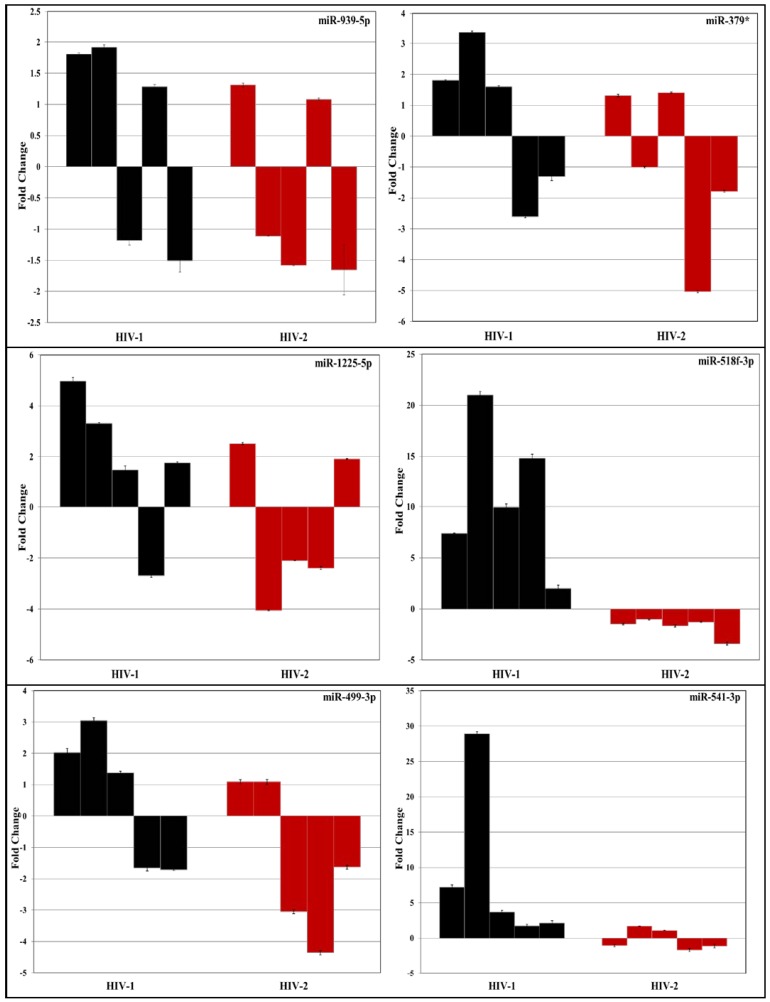

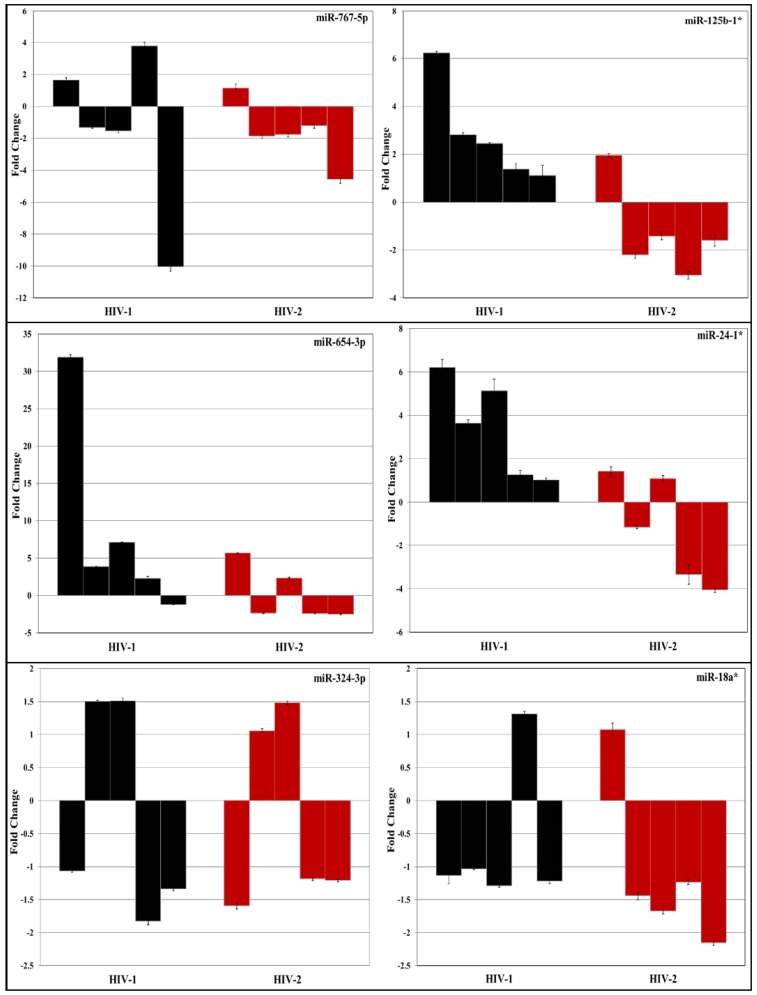

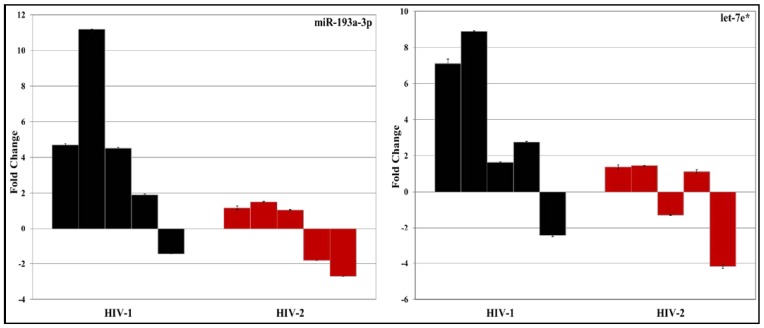
PBMC isolated from independent donors were stimulated and infected with known amounts (5 ng/mL p24/p27 units per 10^6^ cells) of HIV-1 (MN) and HIV-2 (ROD) viruses. Two hours post-infection, the tubes were washed with PBS to remove un-adsorbed virus and cells cultured in complete RPMI-1640 media. Culture supernatants and cells were harvested day 7 post infection. The expression of selected miRNAs was tested in all the independent donor samples using Real-time PCR (RT-PCR). The two-step RT-PCR method was used with TaqMan MicroRNA Reverse Transcription Kit and TaqMan miRNA specific primers (Life Technologies, Inc., Waltham, MA, USA) according to manufacturer’s protocols. RNU44 was used as an internal control for normalization between each donor. Each sample was run in triplicate to ensure accurate fold change estimation. Relative miRNA expression was calculated by the 2^ ^ [−{ΔCt^ ^(infected) − ΔCt (uninfected)}]^ formula. Results are expressed as mean ± standard error of the mean (SEM) of all independent donors’ experiments.

To validate the differentially regulated miRNAs identified using PCR arrays, a subset of 20 differentially expressed miRNAs in HIV-1 and HIV-2 infected PBMC were selected (Figure 3). We tested total RNA isolated from the infected and uninfected PBMC derived from five independent donors for specific miRNAs along with endogenous controls using miRNA specific primers and probes by reverse transcription (RT)-PCR (Figure 4).

**Figure 5:**
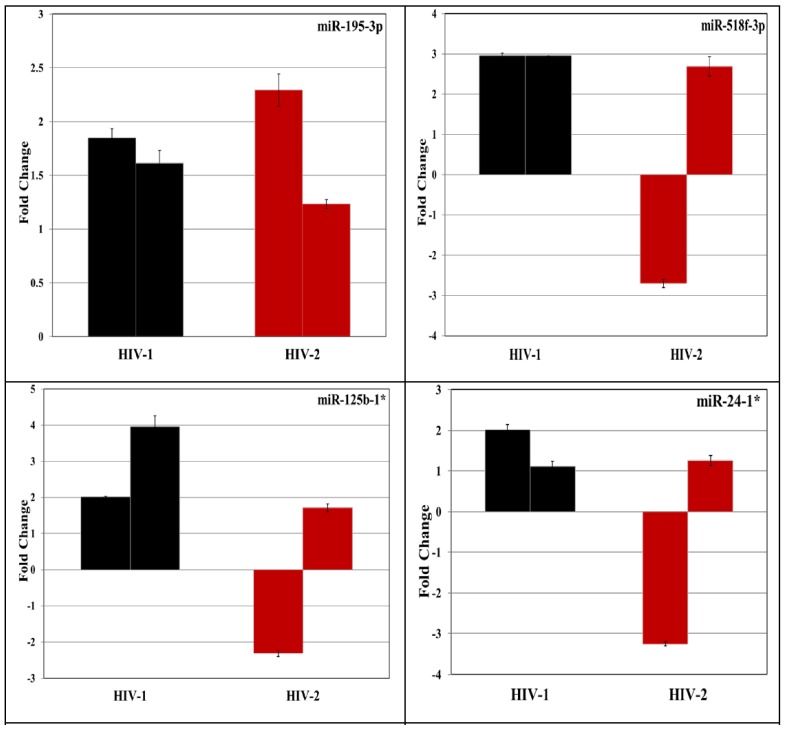

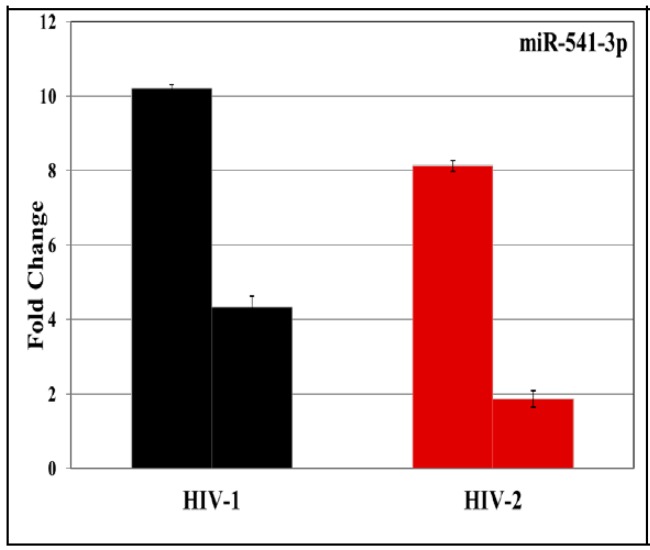
Total RNA was extracted using miRNeasy total RNA isolation kit (QIAGEN) from Jurkat cells infected with HIV-1 (MN) or HIV-2 (ROD) and cultured for 7 days post infection. The expression of selected miRNAs was tested in the independent samples using TaqMan MicroRNA Reverse Transcription Kit and TaqMan miRNA specific primers (Life Technologies) according to manufacturer's protocols. RNU44 was used as an internal control for each sample. Each sample was run in triplicate to ensure accurate fold change estimation. Relative miRNA expression was calculated by the ΔΔCt method. Results expressed as mean ± SEM of all independent donors experiments.

Results indicate that among the twenty differentially expressed miRNAs tested, only nine miRNAs (*hsa-miR 193a-3p*, *hsa-miR 195-3p*, *hsa-miR 499-3p*, *hsa-miR 518f-3p*, *hsa-miR 541-3p*, *hsa-miR 654-3p*, hsa-miR 125b-1*, hsa-miR 24-1* and let-7e*) showed consistent differential expression by independent RT-PCR assay (Figure 4). This could be due to the donor to donor variation. The results obtained from five independent PBMCs infected with HIV-1 and HIV-2 indicates that these miRNAs are differentially regulated across multiple donors in response to infection by HIV-1 or HIV-2. Using RNA isolated from two independent sets of Jurkat cells infected with HIV-1 and HIV-2 (Figure 5) as the template we identified three miRNAs (*hsa-miR 195-3p*, *hsa-miR 518f-3p*, and *hsa-miR-541-3p*) that were consistently upregulated in HIV-1 infected cells. This result is in agreement with our previous RT-PCR results obtained from PBMC, further validating the reproducibility of our PCR array analyses.

### 3.3. Predicted Target Genes of Differential Expressed miRNA

MiRNAs play an important role in transcriptional and posttranscriptional silencing of host mRNAs, multiple mRNA can be potentially regulated by a single miRNA or vice versa [18,38]. We identified putative target genes of the differentially expressed miRNAs using the web-based prediction tool in miRDB database (MirTarget2). We identified that several putative miRNA target genes like Cullin 2 (*CUL2*), member RAS oncogene family (*RAB6A*), *UBE2I*, clathrin heavy chain 1 (*CLTC*), Desmocollin 3 (*DSC3*), Ras Family Small GTP Binding Protein (*RAP1B*), LSM12 homolog (*LSM12*), protocadherin 11 X-linked (*PCDH11X*) and HCLS1 Associated Protein X-1 (*HAX1*) already had predicted interactions with HIV-1 RNA and proteins (Table 3). To further elucidate the functional role of the differentially expressed miRNAs (*miR-195-3p*, *miR-518f-3p*, *miR-541-3p*) in HIV-1 and HIV-2 pathogenesis, we selected ten host genes based on the highest target score for each of the three miRNAs (*miR-195-3p*, *miR-518f-3p*, *miR-541-3p*) for validation.

### 3.4. Validation of miRNA Targets Genes

**Figure 6:**
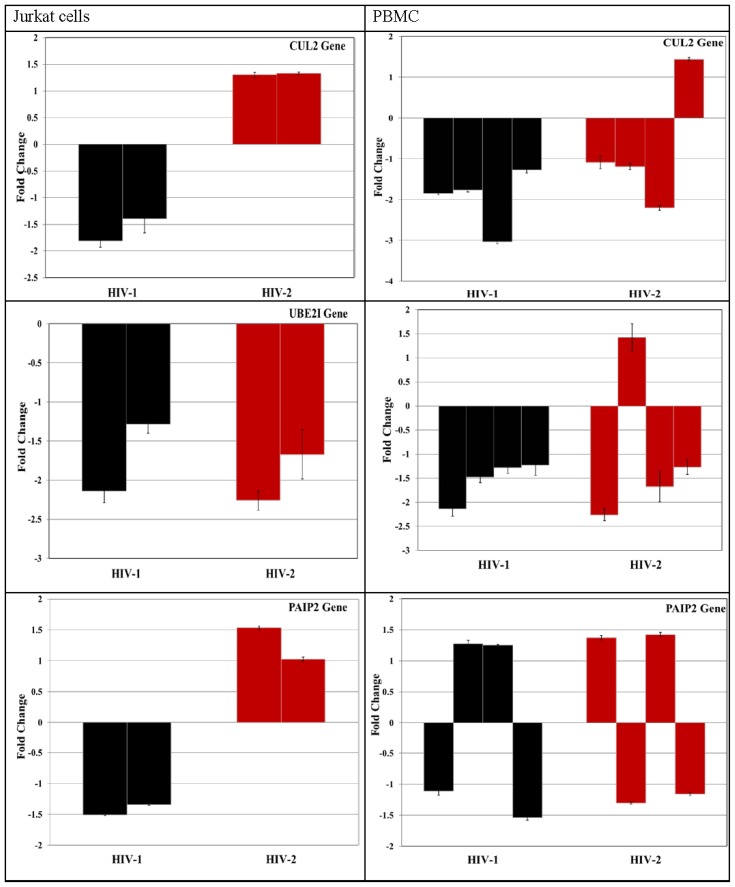

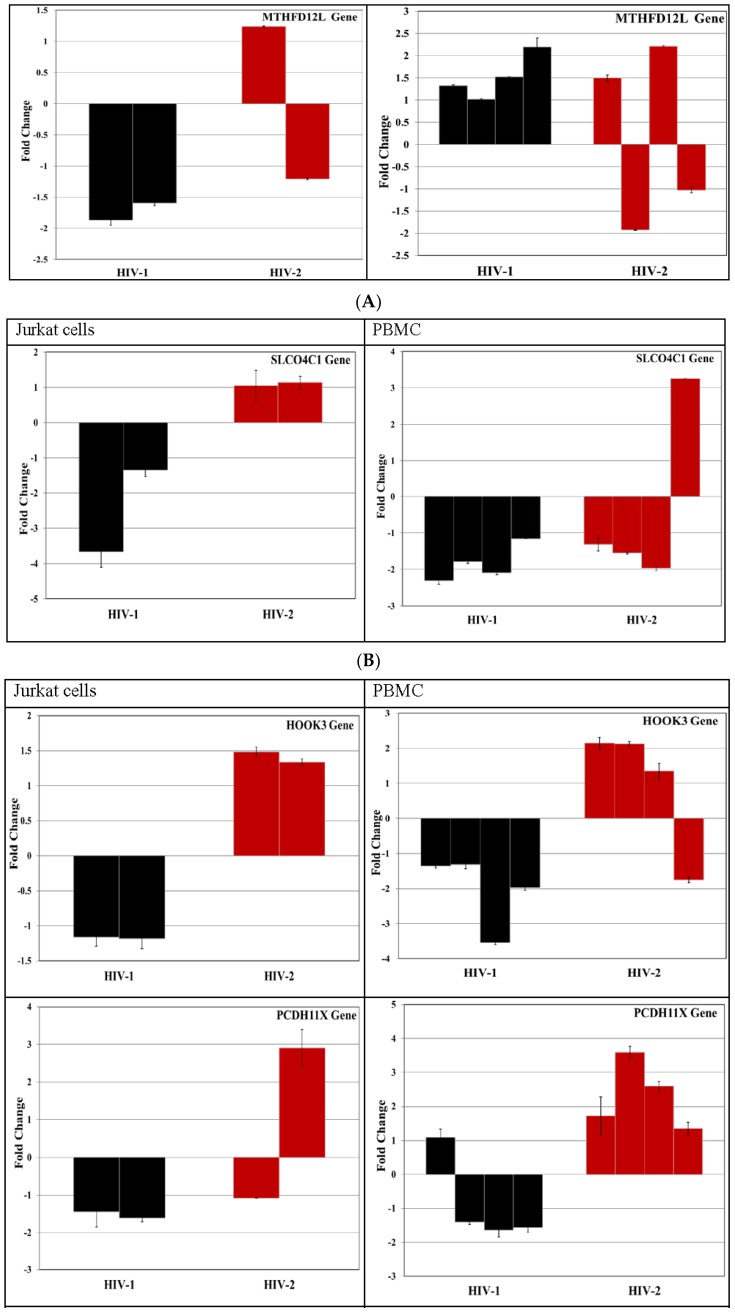

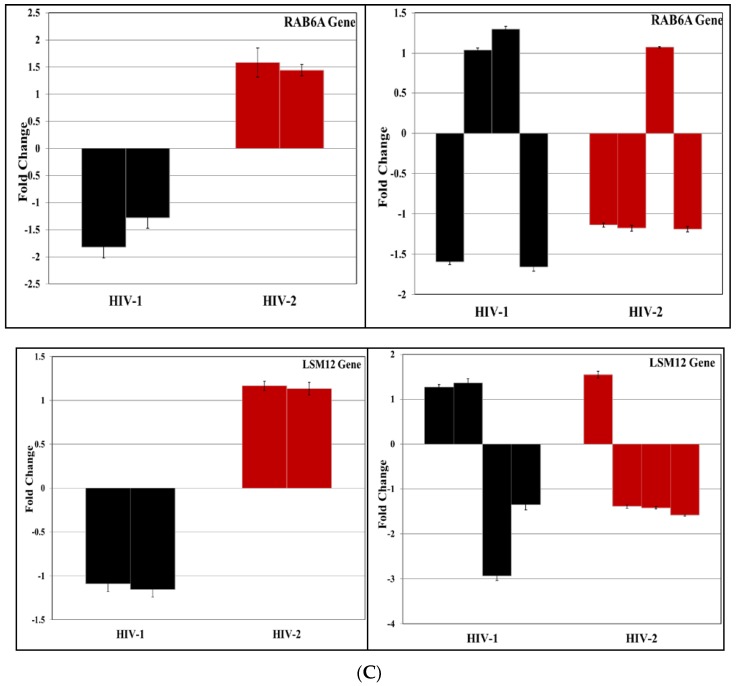
Validation of (**A**) *miR-195-3p*-miRNA host gene; (**B**) *miR-518f-3p* miRNA host gene; (**C**) *miR-541-3p* miRNA host gene targets by RT-PCR in two independent sets of Jurkat cells and PBMC isolated from four independent donors, infected with HIV-1 and HIV-2. The relative expression of the target genes were measured by RT-PCR. Data was analyzed using 2^−∆∆Ct^ method and results were plotted as fold difference of relative expression normalized to the glyceraldehyde-3-phosphate dehydrogenase (*GAPDH*) gene. Results expressed as mean ± SEM of all independent donors’ experiments.

Validation of targeted mRNA is essential because miRNA target prediction algorithms are characterized by high false discovery. To further establish the potential for functional relationship between miRNA and target genes, we validated the targeted mRNA expression with the same sample sets where the miRNAs were differentially expressed. The predicted host target genes were validated in Jurkat cells as well as in PBMC samples. Among the twelve putative mRNA targets that were tested for *miR 195-3p*, we found four genes, *CUL-2*, poly(A) binding protein interacting protein 2 (*PAIP2*), methylenetetrahydrofolate dehydrogenase (NADP+ dependent) 2-like (*MTHFD12L*) and *UBE2I* genes were downregulated in HIV-1 infected Jurkat cells. However, in PBMC only *CUL-2* and *UBE2I* genes were found to be down modulated (Figure 6A). In the same way we validated ten putative mRNA targets each for miRNAs *miR-518f-3p* and *miR-541-3p*. Out of the ten predicted target genes tested for *miR-518f-3p* we found that only one gene, *SLCO4C1* gene was downregulated in HIV-1 infected Jurkat and PBMC cells (Figure 6B). Likewise, the predicted *miR 541-3p* target genes were validated in both Jurkat and PBMC cells. The results showed that in infected Jurkat cells, out of the ten target genes tested, *HOOK3*, *RAB6A*, *LSM12*, and *PCDH11X* were down regulated. However, when these genes were tested in HIV-1 infected PBMC cells only *HOOK3* and *PCDH11X* genes showed consistent downregulation (Figure 6C).

**Figure 7:**
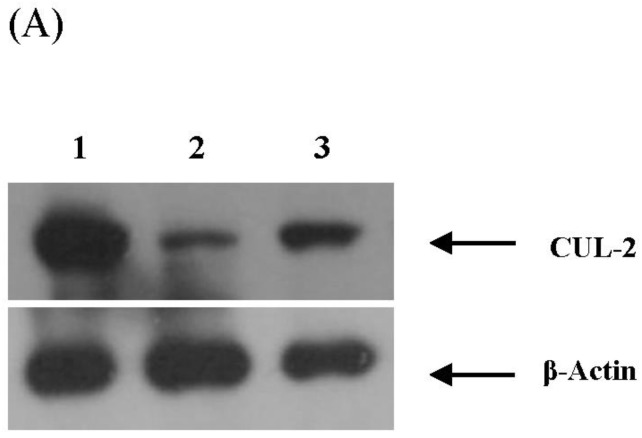

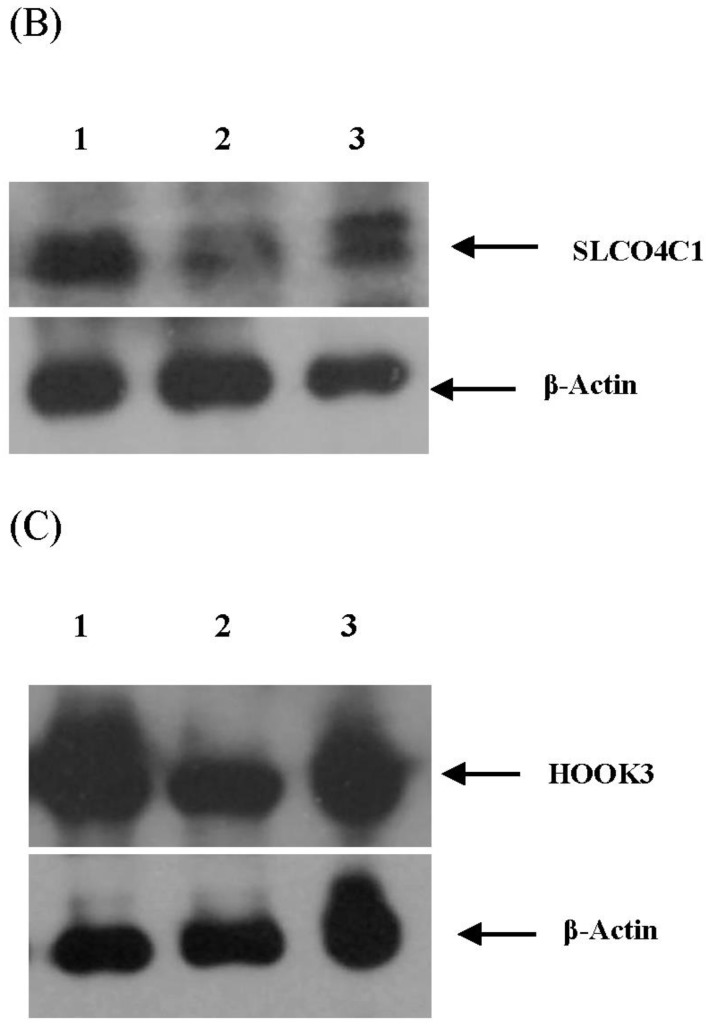
Protein expression validation of (**A**) *miR-195-3p*-miRNA target host gene *CUL-2*; (**B**) *miR-518f-3p* miRNA target host gene solute carrier organic anion transporter family member 4C1 (*SLCO4C1*); (**C**) *miR-541-3p* miRNA target host gene hook microtubule-tethering protein 3 (*HOOK3*) by Western blot in PBMC infected with HIV-1 and HIV-2. Lane 1 is denoted as no virus infection control, Lane 2 is denoted as HIV-1 (MN) infected PBMC and Lane 3 is denoted as HIV-2 (ROD) infected PBMC respectively. The data is representative of three independent experiments.

The expression of target genes at the protein level was validated to further determine the functional relationship between the miRNAs and target genes. Expression levels of selected miRNA target genes were assessed by Western Blotting. The expression of CUL-2 and UBE21 the predicted targets of *miR 195-3p*, SLCO4C1 the predicted target of *miR 518f-3p*, and HOOK3 the predicted target of *miR 541-3p* were determined in cell lysates of PBMCs infected with HIV-1 or HIV-2. The results indicate (Figure 7) that protein expression of CUL-2, SLCO4C1 and HOOK3 were all down modulated, which is in concordance with the real-time PCR data.

## 4. Discussion

HIV-1 and HIV-2 are closely related retroviruses that share considerable similarities in their genome architecture, pathogenesis and clinical outcomes. Both HIV-1 and HIV-2 infections lead to acquired immune deficiency syndrome (AIDS), the main difference observed in HIV-2 infected patients being the relative slower disease progression and greater immunological control of HIV-2 replication [13,14,39]. The exact cause of this phenomenon is not known. In order to understand the molecular basis of the differences in disease progression between HIV-1 and HIV-2, previously we examined differences in apoptotic signaling pathways during infection with either HIV-1 or HIV-2 in Jurkat cells and PBMC. Our studies demonstrated that HIV-1 infection induced greater cell death, activation of apoptosis and autophagy, generation of reactive oxygen species (ROS) and increased the expression of diverse molecules involved in cell signaling [16,40].

HIV infection induces multiple changes in the expression profile of host genes including non-coding RNA species, all of which contribute to host response. In order to delineate the differences in virus- host responses to infection with HIV-1 or HIV-2, we wanted to characterize the expression profiles of host miRNAs in cells infected with HIV-1 and HIV-2. Thus, the differential expression of host miRNA expression profiles was analyzed using the miRNA profiling PCR arrays. 

Previous studies comparing miRNA expression profiles in *in vitro* HIV-1 infected T cell lines, PBMCs and cells from infected patients identified several miRNAs that were differentially regulated after HIV-1 infection or exposure to the virus [11,16,19,20,27,41,42,43,44,45,46]. Studies have shown that cellular miRNA expression upon viral infection contributes to HIV-1 latency in resting T-lymphocytes [47], interferes with Nef protein expression and tat protein function and inhibits gag protein assembly leading to decreased HIV-1 infectivity [16,27,48,49,50,51,52]. In contrast, other studies have reported that upregulation of specific host miRNAs [29,46,53,54] enhances HIV-1 replication. While several studies, including the present study identified many miRNAs that were commonly regulated, a greater variation was noted between these studies [20,27,41,42,55,56]. The differences observed could be due to differences in cell type, patient population and the methodology used to identify miRNAs. Our comparative analysis of miRNA expression profiles using PCR arrays in HIV-1 and HIV-2 infected PBMC indicate that miRNA expression profiles were considerably altered in HIV-1 and HIV-2 infected cells compared to uninfected controls. Analysis of the differentially expressed miRNA between the two groups (HIV-1 and HIV-2 infected PBMC) indicates significant overlap as well as group specific expression of miRNAs (Figure 2). The PCR array results demonstrate that five miRNAs were commonly upregulated and four miRNAs were commonly downregulated in both HIV-1 and HIV-2 infected cells. Similarly, 16 miRNAs were upregulated and 11 miRNAs downregulated in HIV-1 infected PBMC. Eight (8) miRNAs were upregulated and 19 miRNAs were downregulated in HIV-2 infected PBMC cells. Consistent with reports in literature, our study also identified several miRNAs (*hsa-miR 195-3p*, *hsa-miR 335*, *hsa-miR 424*, *hsa-miR 518c*, *hsa-miR 541-3p*, and *hsa-miR 518f-3p*) that were modulated by HIV-1 infection [20]. In addition, our PCR array results identified several miRNA species that were uniquely regulated by HIV-1 and HIV-2 infection. Micro-RNA species *hsa-miR-195-3p*, *hsa-miR-513c* and *hsa-miR-518f-3p* were found to be upregulated in HIV-1 infected cells only. Micro-RNA species *hsa-miR-939-5p*, *hsa-miR-125b-1*, and *hsa-miR 654-3p* were found to be upregulated only in HIV-2 infected cells. MicroRNA species *hsa-miR-548m*, *hsa-193a-3p*, and hsa-miR-148b* were found to be upregulated in both HIV-1 and HIV-2 infected cells. Similarly, several miRNA species were found to be dowregulated only in HIV-1 or in HIV-2 infected cells. Independent validation of the PCR array results identified three miRNAs, *hsa-miR-195-3p*, *hsa-miR-518f-3p*, and *hsa-miR-541-3p* that were consistently upregulated in HIV-1 infected cells.

Some of the differentially expressed miRNAs are associated with a wide variety of cancers and are implicated to play an important role in tumor progression. Studies have demonstrated the involvement of *hsa-miR 195* in cancer. MiRNA *hsa-miR-195* was found to be downregulated in several cancers [16,27,57] and is believed to function as a tumor suppressor by targeting BCL2 [16,57,58]. In addition, studies have demonstrated that the overexpression of *hsa-miR 195* in tumor cells leads to the inhibition of cell proliferation, invasion and migration [46,53,59]. Similar studies have identified that *hsa-miR-541-3p* is associated with the inhibition of tumor cell proliferation by inhibiting telomerase activity and transcription factors involved in Wnt signaling pathway [60]. MiRNA *hsa-miR 541* is also implicated in the growth of HER-2 positive breast cancer cells and acts as a negative regulator in the HER-2 pathway [61]. Similarly, studies have demonstrated that *hsa-miR 193a-3p* downmodulated intestinal inflammation and suppress metastasis of non-small cell lung cancer [62]. While the exact mechanism of how these miRNAs modulate HIV infection is not known, high miRNA expression following HIV-1 infection may target cellular factors that act as host restriction factors and facilitate the infection process and promote viral replication. Among the predicted host factors targeted by the upregulated miRNAs in HIV-1 infected cells, we found *CUL 2*, *UBE2I*, *SLCO4C1*, *HOOK3*, and *PCDH11X* genes were consistently down modulated only in HIV-1 infected cells [17].

The host enzyme CUL 2 belongs to a family of structurally related proteins that are involved in the ubiquitin-dependent protein degradation pathway [63]. Protein ubiquitination plays an important role in a wide variety of cellular processes and in HIV-1 infection. HIV-1 integrase enzyme can be actively degraded by the ubiquitin-proteasome pathway [46]. Recent studies have identified that von Hippel-Lindau binding protein 1 (VBP1) and the cullin 2-based von Hippel-Lindau (VHL) ubiquitin ligase are required for HIV-1 expression post integration. The integrase binding protein VBP1 and CUL2-VHL cooperate in the polyubiquitination and degradation of HIV-1 integrase by the ubiquitin-proteasome pathway, an essential step between viral integration and transcription of viral genes [64]. The host gene, *UBE2I* is an E2 Small Ubiquitin-like Modifier (SUMO) conjugase that adds SUMO residues post-translationally to target proteins [65]. Several studies have reported that UBE2I interacts with HIV-1 viral proteins and plays an important role in viral replication [66]. The knockdown of endogenous UBE2I expression in HIV-1 infected cells caused defects in the trafficking of HIV-1 gag and env proteins resulting in the production of defective virus particles that were not infectious [67].

Our studies identified three miRNAs that were differentially expressed in HIV-1 infected cells. We also observed a direct correlation between the upregulation of the three miRNAs and downmodulation of the cellular genes targeted by these miRNAs. The differentially expressed *hsa-miR-195-3p* targets host genes that modulate cellular proteins which are essential for viral replication and disease progression. Thus modulation of host miRNAs in HIV-1 infected cells can potentially enhance the antiviral responses triggered by HIV-1 entry and infection, resulting in increased resistance to productive infection and decreased production of replication competent virus particles.

## Figures and Tables

**Table 1:** Quantification of human immunodeficiency virus type 1 (HIV-1) (P-24) and 2 (HIV-2) (P-27) antigens.

PBMC	HIV-1 (pg/mL)	HIV-2 (pg/mL)
Donor 1	3148.13	28011.78
Donor 2	4019.29	27121.72
Donor 3	1653.06	24534.10

**Table 2:** Differential miRNA expression in HIV-1 and HIV-2 infected PBMC.

No.	miRNA	Fold Change	
		HIV-1 MN	HIV-2 ROD
1	hsa-let-7a-2*	−1.2169	−6.6374
2	hsa-let-7e*	2.0544	1.4174
3	hsa-let-7i*	−1.3374	−2.2122
4	hsa-miR-18a*	−1.1164	−6.7632
5	hsa-miR-105*	−1.4353	−2.4315
6	hsa-miR-148b*	2.5483	2.4489
7	hsa-miR-192*	−1.5557	−2.0177
8	*hsa-miR-193a-3p*	2.7988	2.2536
9	*hsa-miR-195-3p*	3.1952	−1.9213
10	*hsa-miR-1179*	−1.1205	−2.7279
11	*hsa-miR-1206*	2.1144	−1.0175
12	*hsa-miR-1251*	−1.4678	−2.3088
13	hsa-miR-125b-1*	−5.5759	3.3442
14	*hsa-miR-1225-5p*	−1.21	−5.85
15	hsa-miR-23b*	−1.0803	−2.0489
16	hsa-miR-24-1*	−1.1715	−3.0804
17	hsa-miR-25*	−2.3575	1.2734
18	hsa-miR-26a-2*	−2.0392	−2.0489
19	hsa-miR-27a*	−2.9617	1.8709
20	hsa-miR-202*	−1.1892	−2.8631
21	*hsa-miR-217*	−2.1554	−1.3649
22	hsa-miR-218-1*	−1.7084	−2.3989
23	*hsa-miR-220a*	2.4943	1.5979
24	hsa-miR-34a*	−2.2859	−1.3974
25	*hsa-miR-324-3p*	−2.0879	1.4893
26	hsa-miR-335*	−2.2532	−2.5759
27	hsa-miR-340*	−2.3827	−2.0177
28	hsa-miR-374a*	−2.2562	−1.6367
29	hsa-miR-379*	2.1035	−1.0755
30	hsa-miR-424*	4.5302	5.0506
31	*hsa-miR-499-3p*	−2.3432	−2.0667
32	*hsa-miR-499-5p*	3.3336	2.9536
33	*hsa-miR-513b*	1.9684	1.2096
34	*hsa-miR-513c*	2.6708	−1.7721
35	*hsa-miR-518f-3p*	3.4019	1.8917
36	*hsa-miR-541-3p*	5.6293	3.5696
37	*hsa-miR-548m*	2.1098	2.0008
38	*hsa-miR-635*	1.1136	−2.3079
39	*hsa-miR-654-3p*	1.8715	2.2779
40	hsa-miR-708*	1.209	−2.021
41	*hsa-miR-767-3p*	1.9017	−1.0177
42	*hsa-miR-767-5p*	2.6795	1.0528
43	*hsa-miR-769-5p*	1.9541	1.0458
44	*hsa-miR-891b*	2.2034	1.1506
45	*hsa-miR-939-5p*	−1.1593	4.6161
46	*SNORD44*	1.0339	1.0843

**Table 3:** List of miRNA with their predictive target genes and interaction with HIV-1.

Target Score ^1^	Gene	HIV-1 Interactions ^2^	
		Protein	Other
***hsa-miR-195-3p***			
99	*CDK6* (cyclin-dependent kinase 6)	-	-
99	*CUL2* (cullin 2)	Vif; p23	-
99	*RAB6A* [RAB6A, member RAS oncogene family]	gp120; gp41	Replication
99	*CLTC* [clathrin, heavy chain (Hc)]	gp120; Nef; p27; Vpr, p15; matrix; pol	-
99	*DSC3* [desmocollin 3]	gp160	-
99	*DENND1B* [DENN/MADD domain containing 1B]	-	-
98	*CADM2* [cell adhesion molecule 2]	-	-
98	*PAIP2* [poly(A) binding protein interacting protein 2]	-	-
98	*UBE2I* [ubiquitin-conjugating enzyme E2I]	gp120; gp41; Pr55(Gag); integrase; p6	-
98	*MTHFD2L* [methylenetetrahydrofolate dehydrogenase (NADP+ dependent) 2-like]	-	-
***hsa-miR-518f-3p***			
87	*VAPB* [VAMP (vesicle-associated membrane protein)-associated protein B and C]	-	-
81	*PRDX6* [peroxiredoxin 6]	-	-
79	RAP1B [RAP1B, member of RAS oncogene family]	-	Replication
79	*SLCO4C1* [solute carrier organic anion transporter family, member 4C1]	-	-
78	*ZNF282* [zinc finger protein 282]	-	-
75	*TOLLIP* [toll interacting protein]	-	-
74	*TSN* [translin]	-	-
73	*MYO10* [myosin X]	-	-
71	*PARP11* [poly (ADP-ribose) polymerase family, member 11]	-	-
70	*HMP19* [HMP19 protein]	-	-
***hsa-miR-541-3p***			
90	*LOC100507421* [transmembrane protein 178-like]	-	-
89	*HOOK3* [hook homolog 3 (Drosophila)]	-	-
89	*TRIM67* [tripartite motif containing 67]	-	-
85	*SLC37A1* [solute carrier family 37 (glycerol-3-phosphate transporter), member 1]	-	-
83	*CACNA1E* [calcium channel, voltage-dependent, R type, alpha 1E subunit]	-	-
83	*LSM12* [LSM12 homolog (S. cerevisiae)]	gp 120	-
82	*CPLX4* [complexin 4]	-	-
82	*PCDH11X* [protocadherin 11 X-linked]		Replication
80	*HAX1* [HCLS1 associated protein X-1]	Rev, p19	-
78	*DDX17* [DEAD (Asp-Glu-Ala-Asp) box polypeptide 17]	Rev, p19; pol	-

^1^ Target score generated from web-based MirTarget2 prediction tool in miRDB database. ^2^ These interactions were generated using the interaction database form National Center for Biotechnology Information (NCBI).
